# Real-World Efficacy and Safety of Dupilumab Use in Japanese Adult Patients with Atopic Dermatitis: A Single-Center, Retrospective, 104-Week, Observational Study

**DOI:** 10.3390/diseases13020044

**Published:** 2025-02-03

**Authors:** Tetsuharu Ikegami, Ken Igawa

**Affiliations:** Department of Dermatology, School of Medicine, Dokkyo Medical University, Shimotsuga-gun, Tochigi 3210293, Japan; tetsu24@dokkyomed.ac.jp

**Keywords:** dupilumab, atopic dermatitis, Japan, interleukin, head and neck region

## Abstract

**Background/Objectives:** Dupilumab is an interlekin-4 receptor antibody that exerts its efficacy by inhibiting the signaling pathway of interleukin-4/interleukin-13, and it is currently used clinically as a highly potent therapeutic for atopic dermatitis. However, there have been few reports on the therapeutic effect of dupilumab using long-term real-world data. To accumulate further real-world data through long-term use of dupilumab, we performed a retrospective study on the courses of patients with atopic dermatitis who were treated with dupilumab for at least 104 weeks in our university hospital. **Methods:** We examined the treatment courses of 30 adult patients. **Results:** Subjective (e.g., itch visual analog scale and Dermatology Life Quality Index) and objective (e.g., Eczema Area and Severity Index) indices and some biomarkers showed improvements over time with dupilumab treatment, even in cases with poor early response to dupilumab treatment. As for the therapeutic effect on anatomical regions, although the therapeutic effect on the head and neck region was weak in the early stages, it improved over time, and at 104 weeks, it showed a therapeutic effect that was comparable to other regions. **Conclusions:** Therefore, our study demonstrated the advantages of prolonged administration of dupilumab in atopic dermatitis.

## 1. Introduction

In recent years, many previously unknown components of the patho-mechanisms of atopic dermatitis (AD) have been determined, and novel treatments based on these mechanisms have been developed [1,2]. Dupilumab is a novel systemic therapy for AD and was approved in 2018 in Japan. More than 6 years have already passed since the approval of dupilumab in Japan. At present, there are many clinical problems with dupilumab that still need to be solved, such as its use, indications, safety for long-term use, and high medical expenses. However, dupilumab not only demonstrates a strong therapeutic effect on skin inflammation, as anticipated, but also effectively alleviates itching [3]. In particular, how dupilumab should be used and whether it should be discontinued after a certain period of use or continued are extremely important issues in the clinical setting. In addition, there are controversial reports regarding the therapeutic effect of dupilumab on skin symptoms of the head and neck [4,5], and there is still much to be studied. Recently, data analyses of cases in which the drug was used for three years have begun to be reported [6,7], and these studies have demonstrated that dupilumab maintains its effectiveness in controlling AD symptoms over time. For instance, a study showed that after 30–36 months of treatment, 80.7% of patients reported adequate disease control, and 75.3% noted significant improvement in itching, a common and distressing symptom of AD [6]. Another study indicated that EASI-75 and EASI-90 were achieved by 91.8% and 77.2% of patients after three years of dupilumab use [7]. However, these studies only show the therapeutic effect of the cases, and there are no reports yet that answer the above questions and considerations regarding optimal use and/or discontinuation of dupilumab. Moreover, although the head and neck symptoms during dupilumab use have been mentioned as side effects of dupilumab [5], ongoing research should be conducted to better understand the underlying mechanisms and to develop effective management strategies for this complication. Therefore, there is still a strong need to accumulate real-world data.

In this study, we report the treatment courses of 30 patients with AD who received dupilumab for at least 104 weeks at our hospital. Our study demonstrates the advantages of prolonged administration of dupilumab in atopic dermatitis.

## 2. Materials and Methods

In our hospital, adult patients with AD who were treated with usual topical treatment for a certain period of time, who did not respond well to treatment, who met the objective evaluation criteria for the administration of dupilumab in Japan (an investigator’s global assessment [IGA] score >3, body surface area [BSA] >10%, and an Eczema Area and Severity Index [EASI] score >16, or an EASI score of the head and neck region >2.4) [8], and who were treated with dupilumab after treatment options were initially assessed. Among these patients, 30 patients who had been treated with dupilumab for at least 104 weeks were included in this study. This retrospective study was approved by the ethics review board of Dokkyo Medical University (R-62-5J).

All patients had objective and subjective severity scores recorded at the start of dupilumab treatment and periodically throughout the course of treatment. As a routine clinical practice, periodic peripheral blood tests were performed to measure serum immunoglobulin E (IgE) antibody concentrations, serum thymus and activation-regulated chemokine (TARC) concentrations, and eosinophil count. The objective severity evaluation system used the IGA and EASI scores, and the subjective severity evaluation system used the patient-oriented eczema measure (POEM), Dermatology Life Quality Index (DLQI), and itch visual analog scale (VAS).

The EASI score was evaluated by the value itself and by how much the EASI score improved at the start of treatment (the percentage of reduction in EASI score; “EASI50” indicates 50% of the EASI score at the start of treatment).

## 3. Results

### 3.1. Characteristics of the Patients

The characteristics of patients enrolled in this study are shown in Table 1. The average age of the patients with AD was 34.2 years (15–54 years); therefore, all patients were adults (Japanese criteria). There were 19 (63.3%) male patients. Regarding the past history and complications of allergic diseases, there were 20 (66.7%) cases of bronchial asthma, 15 (50%) cases of allergic rhinitis, and 23 76.7%) cases of hay fever. Twenty (66.7%) patients had a family history of allergic disease described above (bronchial asthma, allergic rhinitis, hay fever). None of patients participating in this study received dupilumab monotherapy. Before participating in this study, they had received treatment mainly with various topical drugs, including topical steroids, calcineurin inhibitors, and JAK inhibitors. Even after participating in this study, the various topical treatments continued to be basically the same.

Prior to the administration of dupilumab, topical steroids were administered to all patients; six patients received oral cyclosporine therapy, and three patients received ultraviolet therapy (narrow band-UVB).

The IGA score was IGA3 in 17 (56.7%) patients and IGA4 in 13 (43.3%) patients, and the average EASI score was 27.3 (15–59). The average itch VAS score was 63.9 (31–90/100 mm), the average POEM was 20.2 (10–28), and the average DLQI was 12.7 (1–27).

### 3.2. Changes over Time in Objective Indices (e.g., EASI), Subjective Indices (POEM, Itch VAS, and DLQI), and Serum Markers (IgE, TARC, and Eosinophil Count)

#### 3.2.1. Overall EASI Score

**Figure 1:**
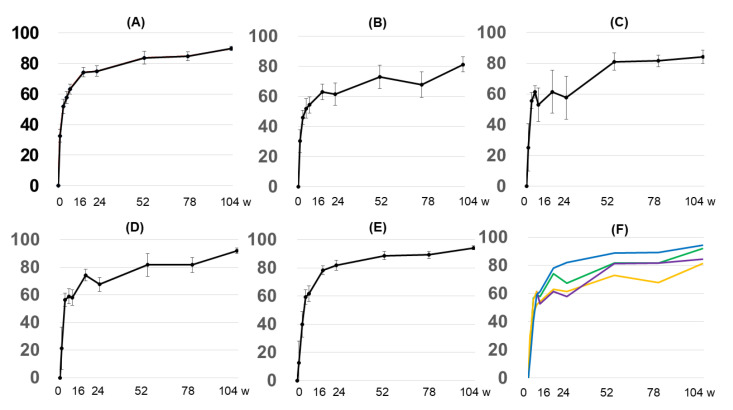
Changes in the percentage of reduction in EASI score over time. (**A**) Overall percentage of reduction in EASI score. (**B**) The percentage of reduction in EASI score in the face, head, and neck. (**C**) The percentage of reduction in EASI score in the trunk. (**D**) The percentage of reduction in EASI score in the upper extremities. (**E**) The percentage of reduction in EASI score in the lower extremities. (**F**) Integrated graph; (**B**) orange, (**C**) purple, (**D**) green, and (**E**) blue. The Y-axis shows the average percentage of reduction in EASI score, and the X-axis shows the number of weeks elapsed.

The average percentages of reduction in EASI score in 30 patients were EASI74.2 at 16 weeks, EASI74.8 at 24 weeks, EASI83.7 at 52 weeks, EASI84.7 at 76 weeks, and EASI89.8 at 104 weeks (Figure 1A). The proportions of patients achieving EASI50, EASI75, EASI90, and EASI100 at week 16 were 80%, 53.3%, 20%, and 0%. The proportions of patients achieving EASI50, EASI75, EASI90, and EASI100 at week 52 were 92%, 84%, 36%, and 12%. The proportions of patients achieving EASI50, EASI75, EASI90, and EASI100 at week 104 were 100%, 100%, 60%, and 16%.

#### 3.2.2. EASI Score by Anatomy

When we investigated an improvement in the anatomy due to treatment, all areas showed good improvement. However, the extremities showed a tendency to improve from the early stage (16, 24, 52, 76, and 104 weeks: EASI82.4, EASI85.6, EASI86.8, EASI89.05, and EASI94.0, respectively), followed by the trunk (16, 24, 52, 76, and 104 weeks: EASI61.5, EASI57.7, EASI81.1, EASI81.6, and EASI84.3, respectively) and the head and neck (16, 24, 52, 76, and 104 weeks: EASI62.9, EASI61.6, EASI73.1, EASI67.9, and EASI81.3, respectively) (Figure 1B–E).

#### 3.2.3. Grouping by Early Treatment Response and EASI Score

**Figure 2:**
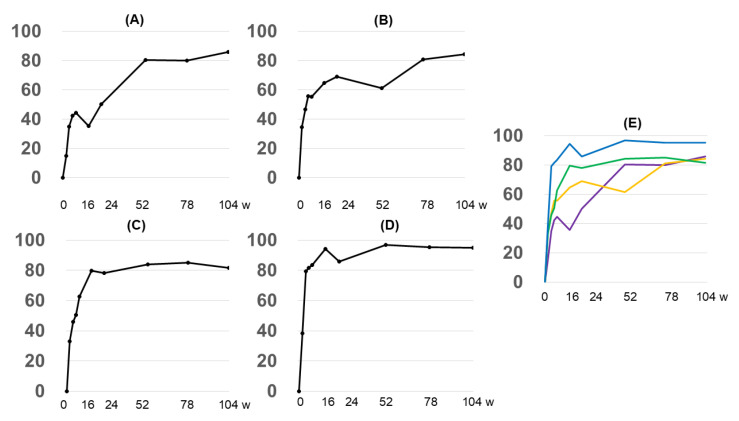
Changes in the EASI clearance rate over time by group based on the EASI clearance rate at 16 weeks. (**A**) <EASI50, (**B**) EASI50-75, (**C**) EASI75-90, and (**D**) >EASI90. (**E**) Integrated graph; (**A**) purple, (**B**) orange, (**C**) green, and (**D**) blue. The Y-axis shows the average percentage of reduction in EASI score, and the X-axis shows the number of weeks elapsed.

The patients were divided into four groups according to the degree of improvement at 16 weeks (<EASI50, EASI50-75, EASI75-90, and >EASI90), and we observed the course in each group. All groups, including the group that did not achieve a favorable improvement trend at 16 weeks (<EASI50 group), achieved an average EASI80 at 104 weeks (Figure 2A–D).

#### 3.2.4. Subjective Indices

**Figure 3:**
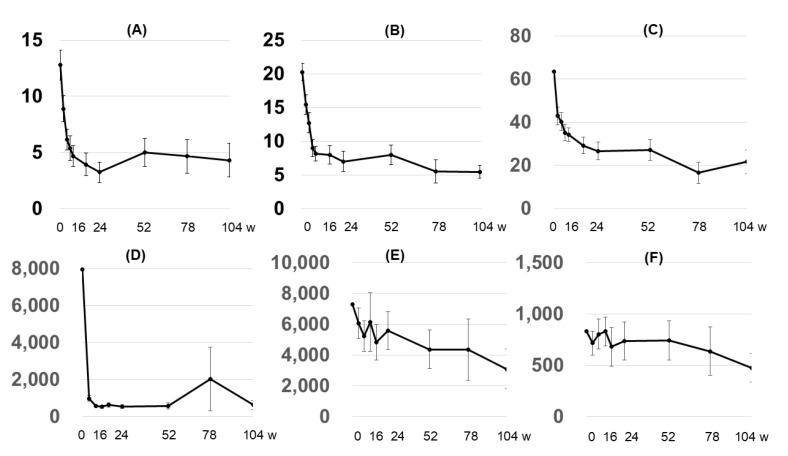
Changes in subjective metrics and biomarkers over time. (**A**) DLQI, (**B**) POEM, and (**C**) itch VAS; (**D**) TARC (pg/mL), (**E**) IgE (IU/mL), and (**F**) eosinophil count (/µL). The X-axis shows the number of weeks elapsed.

The subjective indices, which comprised the itch VAS, POEM, and DLQI, also showed a trend in improvement over time, similar to the EASI score (Figure 3A–C).

#### 3.2.5. Biomarkers

Serum TARC concentrations normalized (<450 pg/mL) immediately after the administration of dupilumab and remained almost unchanged throughout the course. Serum IgE concentrations and the eosinophil count showed a gradual decreasing trend over the course, although this decrease was slower than that for serum TARC concentrations (Figure 3D–E).

### 3.3. Safety Issues

Adverse reactions occurred in nine patients during the course of treatment. There were seven cases of conjunctivitis, one case of herpes zoster, and one case of acne. None of the seven patients with conjunctivitis had any problems with administration of antiallergic eye drops or follow-up. The time of onset of conjunctivitis was variable, ranging from 3 weeks at the shortest to 76 weeks at the longest.

## 4. Discussion

Five years have already passed since dupilumab began to be used in Japan, and various real-world data have been reported [9,10,11]. These reports have shown that dupilumab is effective for the treatment of AD. Our study also shows that dupilumab was effective for treating AD at our hospital.

In this study, the overall EASI score at week 16 was close to EASI75, which was not different from that in a previously reported clinical trial [12]. Interestingly, after 52, 76, and 104 weeks, the degree of improvement in the EASI score gradually increased, and EASI90 was recorded at 104 weeks (Figure 1A). Patients who did not achieve EASI50 at week 16 continued to use dupilumab, and the degree of improvement gradually increased at 52, 76, and 104 weeks. At the final time point of 104 weeks, this group of patients was able to achieve a mean EASI80 (Figure 2A). These findings suggest that the patient’s condition may gradually improve as dupilumab continues to be used. These results were consistent with previous reports of sustained improvement in skin symptoms following long-term use of dupilumab [6,7]. Also, even in groups who do not achieve sufficient therapeutic effects in the early stages of treatment, sufficient therapeutic effects may be achieved through continuous use of dupilumab (Figure 2). There have been no similar reports to date, and we believe that this may have a significant impact on the strategy of AD treatment with dupilumab. In other words, if sufficient therapeutic effects are not observed in the early stages of treatment, it may be possible to continue using dupilumab while observing for a while rather than immediately discontinuing treatment with dupilumab.

Previous studies have reported that there is a difference in an improvement in the skin depending on the anatomical site when using dupilumab [13,14]. In particular, reports on dupilumab have shown that improvements in the condition of the skin on the face, head, and neck are inferior to those on other parts of the body [13,14]. Moreover, Vittrup et al. reported that dupilumab is not expected to be effective for skin symptoms on the head and neck based on their 104-week observation results [15]. On the other hand, therapeutic effects of dupilumab that are not related to the anatomical region have also been reported [16]. Even in reports stating that there is no difference in therapeutic effects depending on the anatomical region, it seems that the therapeutic effects in the head and neck region tend to be lower than in other regions [16]. The appearance of seborrheic dermatitis-like reactions due to this disease has been also discussed [16]. In our study, the percentage of reduction in EASI score at 16 weeks at the head and neck was inferior to that in other sites. However, after this time, as with the other sites, the degree of improvement gradually increased, and an average EASI80 was achieved at 104 weeks. The head and neck region has traditionally been an intractable site in AD [17,18,19], and it may also contain residual telangiectasia as a result of long-term inflammation or topical steroids. There are probably differences in reports regarding therapeutic efficacy, but many researchers may agree that dupilumab is not good for treating skin conditions on the head and neck. However, our study suggests that dupilumab is not less effective for skin symptoms in the head and neck, but in more than a few cases, the effect of this treatment may be delayed owing to the reasons mentioned above. Actually, the exact mechanisms behind dupilumab-associated head and neck dermatitis are not fully understood. In this study, we also did not investigate the mechanisms. Considering the hypothesis of *Malassezia* involvement [13,14], further research is necessary.

In this study, subjective indices and biomarkers showed a clear trend of improvement over time, which is consistent with previous reports [9,11]. Although dupilumab has a significant impact on reducing inflammatory biomarkers in atopic dermatitis (IgE, TARC, etc.), ongoing research is focused on understanding how these biomarkers can predict treatment responses and improve patient outcomes. For instance, elevated levels of the TH17 cell-related cytokine CXCL2 have been associated with better treatment outcomes [20]. Additionally, biomarkers like CD25 and soluble interleukin-2 receptor alpha (sIL-2Rα) have been identified as relevant in assessing treatment efficacy [21].

No serious side effects were observed in the 30 patients who received dupilumab for at least 104 weeks. Concerning conjunctivitis, a systematic review and meta-analysis revealed that dupilumab users have a significantly higher risk of developing conjunctivitis compared to those receiving placebo, with a risk ratio of 1.89 overall. Specifically, patients with atopic dermatitis exhibited an even higher risk (risk ratio of 2.43) compared to those with other indications [22,23]. In our present study, conjunctivitis was observed in 7 of 30 (23.3%) patients, which is similar to previous reports [9,11,12], and those conditions were not serious.

## 5. Conclusions and Limitations

This study was retrospective and had a small sample size. Therefore, our results and their interpretation are limited. Also, the method of selecting patients in this study may have introduced selection bias, because the criteria for inclusion were not well-defined; for instance, previous treatment methods, medical history, and the presence or absence of complications were not taken into consideration. However, we found that the continuous use of dupilumab improved skin symptoms for at least 104 weeks, and that the degree of improvement gradually increased. These real-world clinical results should be helpful for clinicians prescribing dupilumab.

## Figures and Tables

**Table 1:** Characteristics of AD patients enrolled in this study.

Characteristic	Mean
Age, y	34.2
F/M (*n*)	11/19
IGA *	IGA3: 17 (*n*) IGA4: 13 (*n*)
EASI score	27.3
Athma (*n*)	20
Rhinitis (*n*)	15
Pollinosis (*n*)	23
DLQI	12.8
POEM	20.2
Itch VAS	63.5
Serum TARC level (pg/mL)	7945.9
Serum IgE level (IU/mL)	7296
Eosinophil count (/µL)	830

* IGA3: moderate severity, IGA4: severe severity.

## Data Availability

The original contributions presented in the study are included in the article; further inquiries can be directed to the corresponding author.

## Abbreviations

The following abbreviations are used in this manuscript:

EASI	Eczema Area and Severity Index
IGA	investigator’s global assessment
BSA	body surface area
POEM	patient-oriented eczema measure
DLQI	Dermatology Life Quality Index
VAS	visual analog scale
TARC	thymus and activation-regulated chemokine
IgE	immunoglobulin E
